# Comparison of culture and qPCR for the detection of *Pseudomonas aeruginosa *in not chronically infected cystic fibrosis patients

**DOI:** 10.1186/1471-2180-10-245

**Published:** 2010-09-24

**Authors:** Pieter Deschaght, Petra Schelstraete, Guido Lopes dos Santos Santiago, Leen Van Simaey, Filomeen Haerynck, Sabine Van daele, Elke De Wachter, Anne Malfroot, Patrick Lebecque, Christiane Knoop, Georges Casimir, Hedwige Boboli, Frédéric Pierart, Kristine Desager, Mario Vaneechoutte, Frans De Baets

**Affiliations:** 1 Laboratory for Bacteriology Research (LBR), Ghent University Hospital, Ghent University, Ghent, Belgium; 2 Cystic Fibrosis Centre Ghent, Ghent University Hospital, Ghent, Belgium; 3 Cystic Fibrosis Centre, Universitair Ziekenhuis Brussel, Brussels, Belgium; 4 Cystic Fibrosis Centre, Cliniques St Luc, University of Louvain, Brussels, Belgium; 5 Cystic Fibrosis Centre ULB, Hôpital Universitaire des Enfants Reine Fabiola, Erasme University Hospital, Brussels, Belgium; 6 Cystic Fibrosis Centre Liege, CHR La Citadelle and CHC Espérance, Liege, Belgium; 7 Cystic Fibrosis Centre Antwerp, University Hospital Antwerp, Antwerp, Belgium

## Abstract

**Background:**

*Pseudomonas aeruginosa *is the major respiratory pathogen causing severe lung infections among CF patients, leading to high morbidity and mortality. Once infection is established, early antibiotic treatment is able to postpone the transition to chronic lung infection. In order to optimize the early detection, we compared the sensitivity of microbiological culture and quantitative PCR (qPCR) for the detection of *P. aeruginosa *in respiratory samples of not chronically infected CF patients.

**Results:**

In this national study, we followed CF patients during periods between 1 to 15 months. For a total of 852 samples, 729 (86%) remained *P. aeruginosa *negative by both culture and qPCR, whereas 89 samples (10%) were positive by both culture and qPCR.

Twenty-six samples were negative by culture but positive by qPCR, and 10 samples were positive by culture but remained negative by qPCR. Five of the 26 patients with a culture negative, qPCR positive sample became later *P. aeruginosa *positive both by culture and qPCR.

**Conclusion:**

Based on the results of this study, it can be concluded that qPCR may have a predictive value for impending *P. aeruginosa *infection for only a limited number of patients.

## Background

Cystic fibrosis (CF) is one of the most common genetic disorders, caused by mutations in the CFTR gene, coding for the Cystic Fibrosis Transmembrane Conductance Regulator (CFTR) protein [1]. Mutations in this gene lead to inactivity of the CFTR protein and/or reduced expression of the protein at the cytoplasmic membrane [2]. Improper functioning of the CFTR results in the production of viscous mucus and in a defective innate immunity [2,3]. The reduced functionality of the mucociliary system and the ongoing inflammation result in an increased sensitivity of the CF airways to infection by bacterial pathogens, of which *Pseudomonas aeruginosa *and *Staphylococcus aureus *are the most important. Chronic lung infection with *P. aeruginosa *is a major cause of morbidity and mortality among the CF patients [4]. It is now well-established that early aggressive antibiotic treatment of new infection with *P. aeruginosa *is successful in postponing chronic infection. Hence, it is important to detect new infection with *P. aeruginosa *as early as possible so that eradication treatment can be started as soon as possible [5-7]. Currently, routine detection and identification of *P. aeruginosa *in respiratory samples is done by conventional methods such as culture and biochemical characteristics. Misidentification can occur due to the variable phenotypic characteristics of this species [8]. Moreover, the sensitivity of culture might be limited, especially when compared to DNA amplification based techniques. Thus far, however, only one group has compared both approaches in a long term study for early detection of *P. aeruginosa *from CF patients [9].

In this national study, we followed CF patients during periods between 1 to 15 months and we compared the sensitivity of conventional culture techniques with qPCR for the detection of *P. aeruginosa *in the respiratory samples from CF patients, not chronically infected by *P. aeruginosa*.

## Methods

### Patients and sampling

From January 2008 until May 2009, sputum, nasopharyngeal or throat swab samples were routinely collected from 397 CF patients attending all but one of Belgian CF-centres, i.e. Ghent University Hospital (UZG, Ghent), Universitair Ziekenhuis Brussel (UZB, Brussels), St Luc University Hospital (UCL, Brussels), Queen Fabiola Children's University Hospital and Erasme University Hospital (ULB, Brussels), Antwerp University Hospital (UZA, Antwerp), CF Center Liege (CHC - CHR, Liege). Patients were seen every three months and sputum or nasopharyngeal aspirate/throat swab samples were cultured at every visit. Nasopharyngeal aspirates/throat swab samples were collected in case the patients could not expectorate. All 397 included patients, (median age: 14 years, range: 1-53 years), were considered as *P. aeruginosa *free and not chronically infected according to the criteria used by the different Belgian CF centers, i.e., the European Consensus criteria [10] or those defined by Lee *et al. *[11]. For the 252 patients with at least two respiratory samples (median: 3 samples, range: 2-11 samples), the median follow-up time was 6 months (range: 1-15 months). Patients with a *P. aeruginosa *positive culture were treated according to the standard antibacterial treatment protocols of each center, patients with only a PCR positive result were not treated.

### Sample processing

After arrival at the Laboratory Bacteriology Research (LBR), sputum and nasopharyngeal samples were liquefied with Sputasol (Oxoid Ltd., Basingstoke, UK) (1:1, vol/vol, 1 h incubation at 37°C). Throat swabs (ESwab, Copan, Brescia, Italy) were vortexed in the liquid transport medium present in the Eswab tube. For microbiological culture, samples were immediately processed after arrival. For qPCR, at least 200 μl of each sample was stored at -80°C prior to DNA-extraction.

### Culture and identification of the bacteria

Fifty μl of the samples were inoculated onto MacConkey Agar plates (Becton Dickinson, Erembodegem, Belgium) and 100 μl into 4 ml Cetrimide Broth (Fluka Biochemika, Buchs, Switzerland) and incubated for at least 24 h at 37°C at ambient atmosphere before examination. Cetrimide Broth was subcultured by inoculating 50 μl onto a Sheep Blood Agar plate (Becton Dickinson), which was also incubated for at least 24 h at 37°C before examination.

After a maximum of 5 days incubation, lactose negative colonies on MacConkey Agar were picked, subcultured onto a 5% Sheep Blood Agar plate (Becton Dickinson) and identified using tDNA-PCR [12].

### DNA-extraction

Before DNA-extraction, respiratory samples were pre-incubated with proteinase K, i.e. incubation of 200 μl of each sample during 1 h at 55°C in 200 μl proteinase K buffer (1 mg/ml proteinase K, 0.5% SDS, 20 mM Tris-HCl, pH 8.3) with vortexing every 15 min. DNA was extracted using the protocol Generic 2.0.1 on the bioMérieux easyMAG Nuclisens extractor (bioMérieux, Marcy-l'Etoile, France). Final elution volume was 110 μl. This DNA-extraction protocol had been shown previously to be the most sensitive of five different methods [13].

### Quantitative PCR

Quantitative PCR (qPCR), targeting the *oprL *gene (NP_249664), was performed using primers PAO1 A (5' CAGGTCGGAGCTGTCGTACTC 3') and PAO1 S (5' ACCCGAACGCAGGCTATG 3') and hydrolysis probe *oprL *TM (5' FAM-AGAAGGTGGTGATCGCACGCAGA-BBQ 3'), manufactured by TIB Molbiol (Berlin, Germany), as described previously [13]. The reaction mixture contained 4 μl of the LightCycler TaqMan Master mix (Roche, Basel, Switzerland), 0.5 μM of each primer, 0.1 μM of the hydrolysis probe, and 5 μl of DNA extract. The final reaction volume was made up to 20 μl by adding water. Cycling was performed on the LightCycler 1.5 (Roche) with an initial hold of 10 min at 95°C, 45 cycles at 95°C for 10 s, at 55°C for 30 s and at 72°C for 1 s.

Using qPCR, the concentration of *P. aeruginosa *in the respiratory sample is determined as the cycle number whereby the fluorescence signal intensity crosses the detection threshold. This value is expressed as the quantification cycle (C_q_). The number of cycles is inversely correlated to the concentration of *P. aeruginosa *in the sample, e.g. a high cycle number indicates a low the initial concentration of *P. aeruginosa *in the sputum.

### Quality control of culture positive, PCR negative samples

To exclude PCR inhibition as an explanation for the PCR negative, culture positive samples, the PCR mix, containing the DNA extract of the sample, was spiked with an internal amplification control (IAC), as described by Khot *et al. *[14]. Briefly, 10^5 ^Jelly Fish oligonucleotides (105 bp) (IAC-oligo), 0.4 μM forward primer (IAC fw) and 0.4 μM reversed primer (IAC rev) primers were added to the reaction mix, and a separate qPCR experiment, using the SybrGreen kit, was carried out with primers hybridizing to the target DNA. When compared to a set of control samples, i.e. culture and qPCR *P. aeruginosa *positive samples to which the same amount of IAC had been added, the PCR was considered as inhibited by (the DNA extract of) the sample, when an increase of 3 C_q_s could be observed.

To exclude that PCR negativity was due to primer mismatch with the *oprL *gene of the *P. aeruginosa *isolates for culture positive, PCR negative samples, *oprL *PCR was carried out on DNA extracted from the *P. aeruginosa *isolates, cultured from the same samples.

### Ethics

The study was approved by the ethics committee from Ghent University Hospital (project nr. 2007/503). Written informed consent was obtained from the patients > 18 years, or from the parents for the children.

### Statistical analysis

Differences in C_q _values were examined using the Mann-Whitney U test and p values of < 0.05 were considered as significant.

## Results

A total of 852 samples was obtained from 397 not chronically infected CF patients, from six out of the seven Belgian cystic fibrosis centres. Of these, 729 samples (86%) from 307 patients remained *P. aeruginosa *negative by culture and by *P. aeruginosa *specific qPCR and 89 samples (10%) from 64 CF patients were both *P. aeruginosa *culture and qPCR positive (Additional File 1, Table S1). For 11 of the 89 samples (12%), only one culture method was positive, i.e. six times only MacConkey, five times only Cetrimide Broth. For these samples, the mean qPCR C_q_-value was 28.6, while for the samples positive by both culture methods, the mean C_q _value was 26.4 (Table 1) (p > 0.05, not significant).

**Table 1:** Comparison of the sensitivity of detection by qPCR and culture

Number of samples	MacConkey Agar	Cetrimide Broth	qPCR C_q _value (range, SD)
78	**+**	**+**	26.4 (17-32, 4.3)

6	**+**	**-**	29.8 (25-32, 2.7)

5	**-**	**+**	27.3 (22-32, 4.3)

26	**-**	**-**	31.7 (20-34, 3.2)

2	**+**	**-**	NA

3	**-**	**+**	NA

5	**+**	**+**	NA

729	**-**	**-**	NA

NA: no amplification, SD: standard deviation

Twenty-six samples (3%), obtained from 26 CF patients, were culture negative but qPCR positive (Additional File 1, Table S2). False positivity due to cross reaction with other CF associated bacterial species could be excluded because the specificity of the primer set had been tested and confirmed on a broad set of common CF pathogenic species [13].

For 23 of these 26 patients, at least one follow-up sample was obtained. Five of these became *P. aeruginosa *culture positive, of which four after a mean lag time of 3.5 months (range: 2-5 months)(Additional File 1, Table S2, samples nr. 7, 19, 21, 23) and a fifth patient after a lag time of nine months after the first qPCR positive sample (Additional File 1, Table S2, sample nr. 8). The latter patient had in between two culture negative, qPCR negative samples. Three other qPCR positive, culture negative patients (Additional File 1, Table S2, samples nr. 3, 16, 22) had a previous sample that was *P. aeruginosa *culture and qPCR positive (mean lag time 4.3 months, range 3-5 months). The follow-up samples of these three patients were culture and qPCR negative. The average qPCR C_q _value (31.7) for these 26 samples was significantly higher, compared with the C_q _value of culture and qPCR positive samples (26.4) (Table 1) (p < 0.001).

Ten samples, obtained from 9 patients, were *P. aeruginosa *culture positive, but qPCR negative (Additional File 1, Table S3). For five of these ten samples (50%), only one of the culture media yielded a positive result, i.e. three samples remained negative on MacConkey Agar and two sample in Cetrimide Broth. For all these culture positive, PCR negative samples, PCR inhibition could be excluded. Primer mismatch could also be excluded, because the cultured *P. aeruginosa *isolates were *oprL *qPCR positive. At least one follow-up sample could be obtained for five of these patients, and for three the follow-up sample(s) was/were culture and qPCR negative, whereas for two patients the follow-up sample(s) was/were culture and qPCR positive.

When taking culture as the gold standard, the PCR had a sensitivity of 90%, a specificity of 85%, a positive predictive value of 77% and a negative predictive value of 99%.

For the samples with a dissimilar culture and qPCR result, there was no relation with the presence of other bacterial species isolated from the respiratory samples (data not presented) and there was no linkage with the sample type (data not presented).

## Discussion

Early detection of *Pseudomonas aeruginosa *in respiratory samples of CF patients has become of utmost importance, taking into account that it is now possible to postpone chronic infection with the use of early aggressive antibiotic treatment [5-7]. In most routine microbiology laboratories, microbiological culture is still the mainstay for detection of *P. aeruginosa*. However, other detection methods that might be more sensitive than microbiological culture still need evaluation and validation [15].

Serological testing for *P. aeruginosa *antibodies has been proposed as an alternative to culture for the early establishment of new infection episodes. Several groups reported that anti-*P. aeruginosa *antibodies can be detected prior to *P. aeruginosa *detection by culture and prior to the onset of chronic infection [16-18]. However, in a cross-sectional study, da Silva Filho and colleagues [19] found more patients positive with culture or PCR than with serology.

In this prospective study, we evaluated whether qPCR can improve early detection of *P. aeruginosa *in respiratory samples from CF patients, not yet chronically infected with this organism.

During the last decade, several PCR formats and other molecular methods for the detection of *P. aeruginosa *have been developed [9,20-30]. Some groups found a higher sensitivity of PCR in comparison with culture and/or biochemical tests for the detection of *P. aeruginosa *from respiratory samples of CF patients [9,19], while others found no difference [28] or a lower sensitivity for PCR [23]. In this study, we targeted the *oprL *gene [13,21], previously shown to be a more sensitive gene locus than the exotoxin A locus, when applied to CF patient airway samples [9]. In a previous study [13], we compared five DNA-extraction methods, six (q)PCR formats and three culture techniques to optimize and validate the detection of *P. aeruginosa *in sputum from CF patients. In our hands, using a dilution series of *P. aeruginosa *in sputum, the three culture methods were equally sensitive to each other but also to the combination of the most sensitive DNA extraction method and the most sensitive amplification assay, i.e. probe based qPCR.

Surprisingly, there is at present only one published study in which *P. aeruginosa *detection by culture and by qPCR is compared in a long term study [9]. These authors concluded that PCR detected *P. aeruginosa *on average 4.5 months prior to culture. In our opinion, this conclusion should be interpreted with caution, because also in their study only 5 of the 10 culture negative, PCR positive patients became *P. aeruginosa *culture positive during the follow-up period. It can also be argued whether the cultured strain was identical as the one causing PCR positivity 4-17 months prior to culture positivity, given the long follow-up period and the fact that the average conversion rate to culture positivity among CF patients can be considered as relatively high. Finally, the authors also found 5 culture positive, PCR negative samples, for which PCR might have become positive later on, however no follow-up data were reported. In our study, we found that out of the 26 qPCR positive, culture negative samples, only 5 follow-up samples became also *P. aeruginosa *culture positive, of which one became double positive only in the third follow-up episode after initial PCR positivity. The significantly higher C_q _values of these 26 samples, compared to the Cq values of double positive samples, suggest a low *P. aeruginosa *inoculum in the respiratory sample and may explain why the presence of *P. aeruginosa *was missed by culture. Thus, PCR positivity may have had a predictive value for impending infection in only 5 of the 26 patients, whereas in 21 patients a positive PCR signal became negative again and did not predict a positive culture at the next follow-up sample. For three of the 26 qPCR positive, culture negative patients, the previous sample was *P. aeruginosa *culture and qPCR positive but the follow-up samples were culture and qPCR negative. This may indicate that qPCR still detected DNA of already killed bacteria. Another 10 samples (1%) were *P. aeruginosa *qPCR negative but culture positive. False negativity of the qPCR was not the reason for the negative qPCR result, because qPCR inhibition and primer mismatch could be excluded. Interestingly, for 5 of these 10 patients, there was discordance between both culture techniques, suggestive for borderline detection by culture and thus a low inoculum of the pathogen. Such discordance between culture results was observed in only 11 out of 89 qPCR positive samples.

For many samples with discordant qPCR and culture results, a low bacterial inoculum may be the explanation. Based on our results in this study and a previous study [13], both approaches have comparable sensitivity, and at low inocula both may be at the border of their detection limit. In addition, at low inocula the distribution of the bacteria in the sample may be more uneven and because we used different parts of each sample to perform qPCR respectively culture, randomization may have influenced the qPCR and/or culture result negatively. The presence of a low inoculum can be concluded from the significantly higher Cq values of qPCR positive/culture negative samples, compared to the qPCR positive/culture positive samples and from the fact that cultures were positive for only one of both media used in 5 out of 10 qCPR negative/culture positive samples. Possibly other factors, such as sample type, the presence of other bacterial species or the genotype of the *P. aeruginosa *isolate might differentially influence the ease with which *P. aeruginosa *can be detected by culture versus qPCR. Further research is warranted on a larger set of samples with discordant qPCR - bacterial culture results to determine the influence of some of these factors.

## Conclusions

The present study indicates that the currently used routine culture techniques perform equally well as DNA amplification techniques for detection of *P. aeruginosa *in respiratory samples of CF patients, not chronically infected with *P. aeruginosa*. Looking at it from a different angle, qPCR was both sensitive and specific compared with a gold standard of culture.

These data, gathered on clinical samples, confirm the results of our previous laboratory study in which culture methods were equally sensitive to the combination of the most sensitive DNA extraction method and the most sensitive amplification assay, i.e. probe based qPCR [13].

Therefore, we may conclude that for this study, based on a large amount of patients and samples, qPCR for *P. aeruginosa *may have a predictive value for impending *P. aeruginosa *infection in only a limited number of cases.

## Authors' contributions

MV, FDB, SVD, PS and PD conceived the study and designed the experiments. MV, FDB, PD, PS, SVD wrote the manuscript. PD, LVS, GLdSS performed the experiments. Authors from other universities provided patient samples and helped with the manuscript discussion. All authors have read and approved the final manuscript.

## Supplementary Material

Additional file 1 **Table S1: Overview of the culture positive and qPCR positive samples. Table S2: Overview of the culture negative and qPCR positive samples. Table S3: Overview of the culture positive and qPCR negative samples**. Overview of all samples with at least a *P. aeruginosa *positive qPCR or a *P. aeruginosa *positive culture result. Click here for file
