# Supplementary material for: Comparison of culture and qPCR for the detection of Pseudomonas aeruginosa in not chronically infected cystic fibrosis patients
Source: BMC Microbiol. 2010 Sep 24;10:245. doi: 10.1186/1471-2180-10-245 (PMC2949703; doi:10.1186/1471-2180-10-245)
Supplement: Additional file 1 — Table S1: Overview of the culture positive and qPCR positive samples. Table S2: Overview of the culture negative and qPCR positive samples. Table S3: Overview of the culture positive and qPCR negative samples. Overview of all samples with at least a P. aeruginosa positive qPCR or a P. aeruginosa positive culture result. [file 1471-2180-10-245-S1.DOC]

**Additional File 1.**

| **Table S1. Overview of the culture positive and qPCR positive samples** | | | | | | | | | | | | | | | |
| --- | --- | --- | --- | --- | --- | --- | --- | --- | --- | --- | --- | --- | --- | --- | --- |
|  |  |  | |  | |  | |  | **Results for different sampling episodes^c^** | | | | | | |
| **N°** | **Patient-Sample N°** | **Sample type^a^** | | **MC^b^** | | **CB^b^** | | **qPCR** | **I** | **II** | **III** | | **IV** | **V** | **VI** |
| **1** | A013-01 | Unknown | | + | | + | | + | **C+ P+** | / | / | | / | / | / |
| **2** | A015-01 | Unknown | | + | | + | | + | **C+ P+** | / | / | | / | / | / |
| **3** | A016-01 | Unknown | | + | | + | | + | **C+ P+** | / | / | | / | / | / |
| **4** | A017-01 | Unknown | | + | | - | | + | **C+ P+** | / | / | | / | / | / |
| **5** | B044-01 | NPA | | + | | + | | + | **C+ P+** | C- P- | C- P- | | / | / | / |
| **6** | B045-01 | Sputum | | + | | + | | + | **C+ P+** | C- P- | C- P- | | / | / | / |
| **7** | B049-01 | Sputum | | + | | + | | + | **C+ P+** | C+ P+ | / | | / | / | / |
| **8** | B049-02 | Sputum | | + | | - | | + | C+ P+ | **C+ P+** | / | | / | / | / |
| **9** | B054-02 | Sputum | | + | | + | | + | NP | **C+ P+** | C+ P+ | | / | / | / |
| **10** | B054-03 | Sputum | | + | | + | | + | NP | C+ P+ | **C+ P+** | | / | / | / |
| **11** | B068-03 | Sputum | | + | | + | | + | NP | NP | **C+ P+** | | / | / | / |
| **12** | B077-01 | Sputum | | + | | + | | + | **C+ P+** | / | / | | / | / | / |
| **13** | C008-02 | Unknown | | + | | + | | + | NP | **C+ P+** | C- P- | | / | / | / |
| **14** | C026-01 | Unknown | | + | | - | | + | **C+ P+** | / | / | | / | / | / |
| **15** | C028-01 | Unknown | | + | | + | | + | **C+ P+** | C- P- | C- P- | | / | / | / |
| **16** | C029-01 | Unknown | | + | | + | | + | **C+ P+** | C- P+ | C- P- | | / | / | / |
| **17** | C030-01 | Unknown | | + | | + | | + | **C+ P+** | C- P- | C- P- | | / | / | / |
| **18** | C043-03 | Sputum | | + | | + | | + | C- P- | C- P+ | **C+ P+** | | / | / | / |
| **19** | C047-02 | Sputum | | + | | + | | + | C- P- | **C+ P+** | / | | / | / | / |
| **20** | C052-02 | Unknown | | + | | - | | + | C- P- | **C+ P+** | C- P- | | C- P- | / | / |
| **21** | C061-04 | Unknown | | + | | NP | | + | C- P+ | C- P- | C- P- | | **C+ P+** | / | / |
| **22** | C063-01 | Unknown | | + | | + | | + | **C+ P+** | / | / | | / | / | / |
| **23** | C121-01 | NPA | | - | | + | | + | **C+ P+** | / | / | | / | / | / |
| **24** | G009-01 | Sputum | | + | | + | | + | **C+ P+** | / | / | | / | / | / |
| **25** | G018-01 | Sputum | | + | | + | | + | **C+ P+** | C+ P- | C+ P+ | | C+ P- | / | / |
| **26** | G018-03 | Sputum | | + | | - | | + | C+ P+ | C+ P- | **C+ P+** | | C+ P- | / | / |
| **27** | G031-05 | Sputum | | + | | + | | + | C- P+ | NP | C+ P- | | C- P- | **C+ P+** | / |
| **28** | G032-01 | Sputum | | + | | + | | + | **C+ P+** | C- P+ | C- P- | | C- P- | C- P- | / |
| **29** | G045-01 | Sputum | | + | | + | | + | **C+ P+** | NP | C+ P+ | | / | / | / |
| **30** | G045-03 | Sputum | | + | | + | | + | C+ P+ | NP | **C+ P+** | | / | / | / |
| **31** | G046-05 | Sputum | | + | | + | | + | C- P- | C- P- | NP | | C- P- | C+ P+ | **C+ P+** |
| **32** | G048-03 | NPA | | + | | + | | + | C- P- | C- P- | **C+ P+** | | C+ P+ | / | / |
| **33** | G048-04 | NPA | | + | | + | | + | C- P- | C- P- | C+ P+ | | **C+ P+** | / | / |
| **34** | G075-03 | Sputum | | + | | + | | + | C- P- | C- P- | **C+ P+** | | C- P- | C- P- | C- P- |
| **35** | G087-01 | Sputum | | + | | + | | + | **C+ P+** | / | / | | / | / | / |
| **36** | G090-02 | NPA | | + | | + | | + | C- P- | **C+ P+** | C+ P+ | | C+^d^ | / | / |
| **37** | G090-03 | NPA | | + | | + | | + | C- P- | C+ P+ | **C+ P+** | | C+^d^ | / | / |
| **38** | G092-01 | NPA | | + | | + | | + | **C+ P+** | C+ P+ | C+ P+ | | / | / | / |
| **39** | G092-02 | NPA | | + | | + | | + | C+ P+ | **C+ P+** | C+ P+ | | / | / | / |
| **40** | G092-03 | NPA | | + | | + | | + | C+ P+ | C+ P+ | **C+ P+** | | / | / | / |
| **41** | G098-05^e^ | Sputum | | + | | + | | + | C+^d^ | **C+ P+** | C-^d^ | | C-^d^ | C-^d^ | C+ P+ |
| **42** | G098-09^e^ | Sputum | | + | | + | | + | C+^d^ | C+ P+ | C-^d^ | | C-^d^ | C-^d^ | **C+ P+** |
| **43** | G098-10^e^ | Sputum | | + | | + | | + | C+^d^ | C+ P+ | C-^d^ | | C-^d^ | C-^d^ | C+ P+ |
| **44** | G098-11^e^ | Sputum | | + | | + | | + | C+^d^ | C+ P+ | C-^d^ | | C-^d^ | C-^d^ | C+ P+ |
| **45** | G101-01 | NPA | | + | | + | | + | **C+ P+** | C+ P+ | C+ P+ | | / | / | / |
| **46** | G101-02 | NPA | | + | | + | | + | C+ P+ | **C+ P+** | C+ P+ | | / | / | / |
| **47** | G101-03 | NPA | | + | | + | | + | C+ P+ | C+ P+ | **C+ P+** | | / | / | / |
| **48** | G108-01 | NPA | | + | | + | | + | **C+ P+** | C- P- | / | | / | / | / |
| **49** | G112-03 | Sputum | | + | | + | | + | C- P- | C- P- | **C+ P+** | | NP | / | / |
| **50** | G137-01 | Sputum | | + | | + | | + | **C+ P+** | C+ P+ | / | | / | / | / |
| **51** | G137-02 | Sputum | | + | | + | | + | C+ P+ | **C+ P+** | / | | / | / | / |
| **52** | G148-04 | NPA | | + | | + | | + | C- P- | NP | C- P- | | **C+ P+** | / | / |
| **53** | G150-01 | Sputum | | + | | + | | + | **C+ P+** | C- P- | C- P- | | C+ P+ | C- P- | C+ P+ |
| **54** | G150-04 | Sputum | | + | | + | | + | C+ P+ | C- P- | C- P- | | **C+ P+** | C- P- | C+ P+ |
| **55** | G150-06 | Sputum | | + | | + | | + | C+ P+ | C- P- | C- P- | | C+ P+ | C- P- | **C+ P+** |
| **56** | L021-02 | NPA | | - | | + | | + | C- P- | **C+ P+** | C- P- | | C+ P+ | / | / |
| **57** | L021-04 | NPA | | + | | + | | + | C- P- | C+ P+ | C- P- | | **C+ P+** | / | / |
| **58** | L023-01 | Sputum | | + | | + | | + | **C+ P+** | / | / | | / | / | / |
| **59** | L024-01 | Sputum | | + | | + | | + | **C+ P+** | C- P- | C- P- | | C- P- | C- P- | / |
| **60** | L025-01 | Sputum | | + | | + | | + | **C+ P+** | C- P- | C- P- | | / | / | / |
| **61** | L028-01 | BAL | | + | | + | | + | **C+ P+** | C- P- | C- P- | | / | / | / |
| **62** | L029-02 | Unknown | | + | | + | | + | NP | **C+ P+** | C- P- | | / | / | / |
| **63** | V006-01 | Throat Swab | | - | | + | | + | **C+ P+** | C+ P+ | C+^d^ | | C- P- | / | / |
| **64** | V006-02 | Throat Swab | | + | | + | | + | C+ P+ | **C+ P+** | C+^d^ | | C- P- | / | / |
| **65** | V029-02 | Sputum | | + | | + | | + | C- P- | **C+ P+** | C+ P+ | | C- P- | / | / |
| **66** | V029-03 | Sputum | | + | | + | | + | C- P- | C+ P+ | **C+ P+** | | C- P- | / | / |
| **67** | V046-02 | Throat Swab | | + | | + | | + | C- P- | **C+ P+** | C- P- | | / | / | / |
| **68** | V051-02 | BAL | | + | | + | | + | C- P- | **C+ P+** | C+ P+ | | C+ P+ | / | / |
| **69** | V051-03 | Throat Swab | | + | | + | | + | C- P- | C+ P+ | **C+ P+** | | C+ P+ | / | / |
| **70** | V051-04 | Throat Swab | | + | | + | | + | C- P- | C+ P+ | C+ P+ | | **C+ P+** | / | / |
| **71** | V054-02 | Throat Swab | | + | | + | | + | C- P- | **C+ P+** | C+ P+ | | / | / | / |
| **72** | V054-03 | Throat Swab | | + | | - | | + | C- P- | C+ P+ | **C+ P+** | | / | / | / |
| **73** | V066-01 | Sputum | | + | | + | | + | **C+ P+** | C- P+ | C- P- | | C- P- | / | / |
| **74** | V075-01 | Throat Swab | | + | | + | | + | **C+ P+** | / | / | | / | / | / |
| **75** | V076-01 | Sputum | | + | | + | | + | **C+ P+** | / | / | | / | / | / |
| **76** | V084-01 | Sputum | | + | | + | | + | **C+ P+** | / | / | | / | / | / |
| **77** | V095-01 | Throat Swab | | + | | + | | + | **C+ P+** | C- P- | / | | / | / | / |
| **78** | V096-02 | Throat Swab | | - | | + | | + | C- P+ | **C+ P+** | NP | | C- P- | C- P- | / |
| **79** | V104-01 | Throat Swab | | + | | + | | + | **C+ P+** | C+ P+ | / | | / | / | / |
| **80** | V104-02 | Throat Swab | | - | | + | | + | C+ P+ | **C+ P+** | / | | / | / | / |
| **81** | V106-03 | Sputum | | + | | + | | + | C- P- | C- P- | **C+ P+** | | / | / | / |
| **82** | V108-02 | Sputum | | + | | + | | + | C- P- | **C+ P+** | C- P- | | C- P- | / | / |
| **83** | V110-01 | Throat Swab | | + | | + | | + | **C+ P+** | C+ P+ | C+ P+ | | / | / | / |
| **84** | V110-02 | Throat Swab | | + | | + | | + | C+ P+ | **C+ P+** | C+ P+ | | / | / | / |
| **85** | V110-03 | Throat Swab | | + | | + | | + | C+ P+ | C+ P+ | **C+ P+** | | / | / | / |
| **86** | V112-01 | Throat Swab | | + | | + | | + | **C+ P+** | C- P- | C- P- | | / | / | / |
| **87** | V116-03 | Sputum | | + | | + | | + | C- P- | C- P- | **C+ P+** | | / | / | / |
| **88** | V127-02 | Throat Swab | | + | | NP | | + | NP | **C+ P+** | / | | / | / | / |
| **89** | V136-01 | Sputum | | + | | + | | + | **C+ P+** | / | / | | / | / | / |
| **Table S2. Overview of the culture negative and qPCR positive samples** | | | | | | | | | | | | | | | |
|  |  |  |  | |  | |  | | **Results for different sampling episodes^c^** | | | | | | |
| **N°** | **Patient-Sample N°** | **Sample type^a^** | **MC^b^** | | **CB^b^** | | **qPCR** | | **I** | **II** | | **III** | **IV** | **V** | **VI** |
| **1** | A010-03 | Unknown | - | | - | | + | | C- P- | C- P- | | **C- P+** | / | / | / |
| **2** | C005-02 | Unknown | - | | - | | + | | C- P- | **C- P+** | | / | / | / | / |
| **3** | C029-02 | NPA | - | | - | | + | | C+ P+ | **C- P+** | | C- P- | / | / | / |
| **4** | C032-02 | Unknown | - | | - | | + | | C- P- | **C- P+** | | C- P- | / | / | / |
| **5** | C033-02 | Unknown | - | | - | | + | | C- P- | **C- P+** | | C- P- | / | / | / |
| **6** | C036-02 | NPA | - | | - | | + | | C- P- | **C- P+** | | C- P- | C- P- | / | / |
| **7** | C043-02 | Sputum | - | | - | | + | | C- P- | **C- P+** | | C+ P+ | / | / | / |
| **8** | C061-01 | Unknown | - | | - | | + | | **C- P+** | C- P- | | C- P- | C+ P+ | / | / |
| **9** | C064-03 | Unknown | - | | - | | + | | C- P- | C- P- | | **C- P+** | / | / | / |
| **10** | C066-01 | Unknown | - | | - | | + | | **C- P+** | NP | | C- P- | / | / | / |
| **11** | C070-01 | Unknown | - | | - | | + | | **C- P+** | / | | / | / | / | / |
| **12** | C075-02 | NPA | - | | - | | + | | NP | **C- P+** | | C- P- | / | / | / |
| **13** | C122-02 | Unknown | - | | - | | + | | C- P- | **C- P+** | | C- P- | / | / | / |
| **14** | C129-01 | NPA | - | | - | | + | | **C- P+** | C- P- | | / | / | / | / |
| **15** | C132-01 | Unknown | - | | - | | + | | **C- P+** | C- P- | | C- P- | / | / | / |
| **16** | V066-02 | Sputum | - | | - | | + | | C+ P+ | **C- P+** | | C- P- | C- P- | / | / |
| **17** | V070-02 | Sputum | - | | - | | + | | C- P- | **C- P+** | | C- P- | / | / | / |
| **18** | V078-03 | Throat Swab | - | | - | | + | | C- P- | C- P- | | **C- P+** | C- P- | / | / |
| **19** | V096-01 | Throat Swab | - | | - | | + | | **C- P+** | C+ P+ | | C- P- | C- P- | / | / |
| **20** | V109-03 | Throat Swab | - | | - | | + | | C- P- | C- P- | | **C- P+** | C- P- | / | / |
| **21** | G031-01 | Sputum | - | | - | | + | | **C- P+** | C+^d^ | | C+ P- | C- P- | C+ P+ | / |
| **22** | G032-02 | Sputum | - | | NP | | + | | C+ P+ | **C- P+** | | C- P- | C- P- | C- P- | / |
| **23** | G089-01 | NPA | - | | - | | + | | **C- P+** | C+^d^ | | / | / | / | / |
| **24** | G106-01 | Sputum | - | | - | | + | | **C- P+** | C- P- | | C- P- | C- P- | C- P- | / |
| **25** | G146-01 | Sputum | - | | - | | + | | **C- P+** | C- P- | | C- P- | C- P- | / | / |
| **26** | G155-05 | NPA | - | | - | | + | | C- P- | C+ P | | C- P- | C- P- | **C- P+** | C- P- |
| **Table S3. Overview of the culture positive and qPCR negative samples** | | | | | | | | | | | | | | | |
|  |  |  |  | |  | |  | | **Results for different sampling episodes^c^** | | | | | | |
| **N°** | **Patient-Sample N°** | **Sample type^a^** | **MC^b^** | | **CB^b^** | | **qPCR** | | **I** | **II** | | **III** | **IV** | **V** | **VI** |
| **1** | B041-02 | Sputum | + | | + | | - | | C- P- | **C+ P-** | | / | / | / | / |
| **2** | B043-02 | Unknown | + | | + | | - | | C- P- | **C+ P-** | | / | / | / | / |
| **3** | B056-01 | Sputum | - | | + | | - | | **C+ P-** | C- P- | | / | / | / | / |
| **4** | B074-01 | Unknown | + | | + | | - | | **C+ P-** | / | | / | / | / | / |
| **5** | V093-02 | Throat Swab | - | | + | | - | | C- P- | **C+ P-** | | C- P- | C- P- | / | / |
| **6** | G006-02 | Sputum | + | | - | | - | | C- P- | **C+ P-** | | C- P- | / | / | / |
| **7** | G018-02 | Sputum | + | | + | | - | | C+ P+ | **C+ P-** | | C+ P+ | C+ P- | / | / |
| **8** | G018-04 | Sputum | + | | + | | - | | C+ P+ | C+ P- | | C+ P+ | **C+ P-** | / | / |
| **9** | G031-03 | Sputum | + | | + | | - | | C- P+ | C+^d^ | | **C+ P-** | C- P- | C+ P+ | / |
| **10** | G153-01 | Sputum | - | | + | | - | | **C+ P-** | / | | / | / | / | / |

Legend:

a: NPA: Nasopharyngeal aspirate, BAL: Bronchoalveolar lavage

b: MC: MacConkey agar, CB: Cetrimide broth, NP: Not processed.

c: C: culture, P: qPCR. Bold indicates the sample that is considered.

d: Only culture, no qPCR: samples with insufficient material for research testing that were assessed only by routine culture.

e: For this patient (G098), a total of 11 samples were tested. Only samples 4 to 9 are shown. Samples 10 and 11 were also culture and qPCR positive.
